# Human Urine-Derived Stem Cells Alone or Genetically-Modified with FGF2 Improve Type 2 Diabetic Erectile Dysfunction in a Rat Model

**DOI:** 10.1371/journal.pone.0092825

**Published:** 2014-03-24

**Authors:** Bin Ouyang, Xiangzhou Sun, Dayu Han, Shenfu Chen, Bing Yao, Yong Gao, Jun Bian, Yanping Huang, Yadong Zhang, Zi Wan, Bin Yang, Haipeng Xiao, Zhou Songyang, Guihua Liu, Yuanyuan Zhang, Chunhua Deng

**Affiliations:** 1 Department of Urology, the First Affiliated Hospital of Sun Yat-sen University, Guangzhou, People's Republic of China; 2 Reproductive Medicine Center, the Key Laboratory for Reproductive Medicine of Guangdong Province, the First Affiliated Hospital of Sun Yat-sen University, Guangzhou, People's Republic of China; 3 Department of Urology, the Third Affiliated Hospital of Southern Medical University, Guangzhou, People's Republic of China; 4 Shanghai Institute of Andrology, Renji Hospital Affiliated Shanghai Jiao Tong University School of Medicine, Shanghai, People's Republic of China; 5 Department of Urology, Shanghai Tenth People's Hospital, Tongji University School of Medicine, Shanghai, People's Republic of China; 6 Wake Forest Institute of Regenerative Medicine, Wake Forest University, Winston Salem, North Carolina, United States of America; 7 Department of endocrinology, the First Affiliated Hospital of Sun Yat-sen University, Guangzhou, People's Republic of China; 8 Key Laboratory of Gene Engineering of Ministry of Education, School of Life Sciences, Sun Yat-sen University, Guangzhou, People's Republic of China; 9 State Key Laboratory of Biocontrol, School of Life Sciences, Sun Yat-sen University, Guangzhou, People's Republic of China; 10 Verna and Marrs McLean Department of Biochemistry and Molecular Biology, Baylor College of Medicine, Houston, Texas, United States of America; 11 Department of Andrology, Center for Reproductive Medicine, the Sixth Affiliated Hospital of Sun Yat-sen University, Guangzhou, People's Republic of China; Kaohsiung Chang Gung Memorial Hospital, Taiwan

## Abstract

**Aim:**

The aim of this study was to determine the possibility of improving erectile dysfunction using cell therapy with either human urine-derived stem cells (USCs) or USCs genetically-modified with FGF2 in a type 2 diabetic rat model.

**Methods:**

Human USCs were collected from 3 healthy donors. USCs were transfected with FGF2 (USCs-FGF2). Sixty-five SD male rats were divided into five groups (G). A control group of normal rats (G1, n = 10), and four other test groups of type 2 diabetic erectile dysfunction rats: PBS as a negative control (G2, n = 10), USCs (G3, n = 15), lentivirus-FGF2 (G4, n = 15), and USCs-FGF2 (G5, n = 15). Diabetes was induced in the rats via a high fat diet for 28 days and a subsequent intraperitoneal injection of streptozotocin (35 mg/kg). Erectile dysfunction was screened with apomorphine (100 μg/kg). Cell injections in the test groups (G2–G5) occurred directly into the corpora cavernosa. The implanted cells were tracked at 7 days (n = 5 animals/G) and 28 days (n = 10 animals/G) post injection. Mean arterial pressure (MAP), intracavernosal pressure (ICP), expression of endothelial markers (CD31, VEGF and eNOS), smooth muscle markers (desmin and smoothelin), histological changes and erectile function were assessed for each group.

**Results:**

USCs expressed mesenchymal stem cell markers, and secreted a number of proangiogenic growth factors. USCs expressed endothelial cell markers (CD31 and vWF) after transfection with FGF2. Implanted USCs or USCs-FGF2 displayed a significantly raised ICP and ICP/MAP ratio (p<0.01) 28 days after intracavernous injection. Although few cell were detected within the implanted sites, histological and western blot analysis demonstrated an increased expression of endothelial and smooth muscle markers within the cavernous tissue following USC or USC-FGF2 injection.

**Conclusions:**

The paracrine effect of USCs or USCs-FGF2 induced improvement of erectile function in type 2 diabetic rats by recruiting resident cells and increasing the endothelial expression and contents of smooth muscle.

## Introduction

Erectile dysfunction (ED) is a common and distressing complication of diabetes with about 35% to 90% of diabetic men reported to suffer from ED [1]. Type 2 diabetes makes up about 90% of all diabetes cases [2]. Since a similar risk of developing ED was reported between men with type 1 and type 2 diabetes after adjusting for age [3], [4], ED associated with type 2 diabetes is therefore a more prevalent problem. Additionally, ED in men with diabetes is more severe than non-diabetic ED patients [5]. The management of diabetic ED is complex and challenging. The first line medication for ED, phosphodiesterase type 5 inhibitors (PDE5 inhibitors), is currently widely used in ED patient with diabetes [6], [7]; however, the effect of PDE5 inhibitors in diabetic ED is lower than in non-diabetic ED [2]. Endothelial dysfunction and subsequent decreased smooth muscle content may be one of the pivotal reasons for this refractory response of diabetic ED. Therefore new therapeutic strategies targeted towards repairing endothelial function, particularly in the early stage of this disorder, are needed.

Cell-based and gene therapies have become the new cutting-edge therapeutic strategies aimed at discovering a cure for ED [8], [9]. We previously illustrated that vascular endothelial growth factor (VEGF) transfected adipose-derived stem cells (ADSCs) improved erectile function in diabetic rats by enhancing VEGF-stimulated endothelial function and increasing the contents of smooth muscle cells and pericytes [10]. Other mesenchymal stem cells (MSCs), such as rat bone marrow-derived mesenchymal stem cells (BMSCs) [11], VEGF transfected BMSCs [12], VEGF transfected endothelial progenitor cells [13], and autologous ADSCs [14], were reported capable of restoring erectile function in a diabetic animal model. Encouragingly, a clinical study recently reported human umbilical cord blood stem cells generated positive effect on ED in 7 diabetic patients [15]; however, the rigidity of the penis in these patients was insufficient for penetration [15] Therefore a more efficiency strategy is necessary in stem cell therapy for ED.

It has been demonstrated that a subpopulation of stem cells can be easily isolated from human voided urine [16], [17], [18], [19], [20], i.e. urine derived stem cells (USCs). These cells possess the features of a progenitor and are a convenient cell source. USCs display many characteristics of MSCs and are capable of differentiating into multiple cell-lines including endothelial and smooth muscle cells [20]. A major advantage to using USCs is that these cells can be abundantly and noninvasively obtained. In this study, our data displayed that USCs can secrete many proangiogenic growth factors, and hold endothelial differentiation potential, particularly after USCs are genetically modified with fibroblast growth factor 2 (FGF2). These characteristics of USCs indicate a strong potential to improve endothelial function in diabetic ED.

Fibroblast growth factors (FGFs) are multifunctional proteins with a wide variety of functions; they are most commonly mitogens but also have regulatory, morphological, and endocrine effects. The FGF family has been alternately referred to as a “pluripotent” growth factor because of its varied interactions with multiple cell types [21], [22]. One important function of FGF1 and FGF2 is the promotion of endothelial cell proliferation and the physical organization of endothelial cells into tube-like structures in vitro [23]. They induce angiogenesis and enhance the growth of new blood vessels from pre-existing vasculature in vivo [24]. Both FGF1 and FGF2 are more potent angiogenic factors than vascular endothelial growth factor (VEGF) or platelet-derived growth factor (PDGF) [25]. FGF1 expression is mainly contained to the central nervous system while FGF2 is expressed throughout all adult tissues [26], [27]. Additionally, FGF2 is reported to be more essential than VEGF, epidermal growth factor (EGF), or insulin-like growth factor (IGF) for endothelial differentiation of ADSCs [28]. In the absence of VEGF, IGF, or EGF, ADSCs may also display endothelial properties when grown in an FGF2-supplemented medium. We therefore selected FGF2 as a proangiogenic growth factor for this study.

In this study, we determined the proangiogenic paracrine effect of USCs on improving erectile dysfunction using cell therapy with USCs or USCs genetically-modified with FGF2 in a rat model of type 2 diabetic ED.

## Materials and Methods

### Ethics statement

A total of 75 Sprague-Dawley (SD) male rats (200 g∼260 g) were purchased from the Animal Center of Sun Yat-Sen University (Guangzhou, China) and kept under standard laboratory conditions. The rats were given food and ultraviolet-sterilized tap water ad libitum. The animal experimental protocol was approved by the Committee for Animal Care and Use of Sun Yat-sen University. Human urine sample was collected from three healthy adult volunteers. The protocol to use human urine and the informed consent was approved by the Sun Yat-Sen University Health Sciences Institutional Review Board. Written informed consent was obtained from the urine donors.

### Study design

Among 75 rats, 10 rats served as a standard control (G1) and 65 rats were used for the diabetic ED model. Fifty-five out of 65 SD rats successfully developed diabetic ED (G2–G5), while the remaining 10 rats that failed to develop diabetic ED were consequently not used in this study. The 55 rats with diabetic ED were randomly divided into four test groups: intracavernuous injection with PBS (G2, n = 10), with USCs (G3, n = 15), with lentivirus-FGF2 (G4, n = 15), and with USCs expressing FGF2 (USCs-FGF2) (G5, n = 15). Five animals from groups G3–G5 were euthanized at 7 days post injection, and tissues were harvested to track injected cells in the penis. At day 28, the remaining 10 animals in each group were used to determine erectile function and the penis was harvested for histological analysis and expression of endothelial markers (CD31, VEGF, eNOS), and smooth muscle specific markers (desmin and smoothelin) in the corpora cavernosa.

### USCs culture in vitro

A total of 12 sterile voided urine samples were collected from 3 healthy volunteers 23 to 32 years old. After mid and last stream urine was collected, urine samples were centrifuged and cell pellets were washed with PBS. Then cells were plated in 24-well tissue culture plates at about 500 cells per well with a mixed medium composed of keratinocyte-serum free medium and progenitor cell medium in a 1∶1 ratio as we previously reported [16].

### Flow cytometry analysis

In order to analyze cell surface markers, USCs (*p*3) were trypsinized and a cell concentration of 5.0×10^5^ was suspended in pre-chilled PBS containing 1% bovine serum albumin (BSA). Fluorochrome-conjugated antibodies (Table S1) of cell surface markers CD24-FITC, CD29-PE, CD31-FITC, CD34-FITC, CD44-PE, CD45-PE, CD73-PE, CD90-APC, CD105-PE, CD146-PE, SSEA-4-PE and STRO-1-FITC were incubated with USCs for 30 min. IgG1-PE, IgG1-FITC, and IgG1-APC conjugated isotype control antibodies were used to determine background fluorescence. Cells were then washed twice in wash buffer, filtered by a 70 μm cell strainer, and analyzed by flow cytometry (FACS Calibur BD Biosciences, Franklin Lakes, NJ) and FlowJo software.

### Measurement of trophic factors secreted by primary cultured USCs

To determine the paracrine factors secreted by the USCs, a total concentration of 2X10^5^ USCs (*p*3) was seeded and incubated with serum-free DMEM under normal culture condition (20% O_2_, 5% CO_2_, 37°C) for 24 hours. The condition medium was analyzed by human cytokine ELISA plate array I kit (Signosis, Inc, CA) following the manufacturer's instructions. Briefly, 100-ul supernatants from the USCs culture medium were incubated in pre-coated 96 well plates for one hour on a rocking platform at room temperature. After incubation with mixed detection antibody and HRP, the plate with the supernatants of culture USCs was reacted with a chemilumincent substrate and analyzed by a luminometer reader. The relative light unit (RLU) was recorded.

### Transfection with lentivirus

In order to generate entry vectors, FGF2 and GFP genes were cloned into pDONR™221 (Invitrogen) to generate pDown-FGF2 and pDown-GFP by utilizing the Gateway BP recombination reaction following the product instructions. The entry vectors, pUp-promoters (EF1α), pDown-FGF2, and pDown-GFP were recombined into the pDest-puro vector following the protocol for LR recombination reaction to construct expression lentivirus vectors, designated as EF1α-FGF2-GFP [29].

The lentivirus particles were produced by transient cotransfection of 293FT cells with the above expression lentiviral vectors and ViraPower™ Lentiviral packaging mix (Invitrogen) using Fugene 6 transfection reagent (Roche, Indianapolis, USA). The supernatant containing lentivirus was harvested 48∼72 h after transfection and concentrated with Amicon Ultra-15 Centrifugal Filter Units (Millipore) [29], [30]. EF1α-hrGFP Lentivirus particles were generously provided by the Center for Stem Cell Biology and Tissue Engineering, Sun Yat-sen University.

For lentiviral transduction, USCs of passage 2 were dissociated into single cells with 0.25% (w/v) trypsin and replated onto fresh USC medium contain FGF2 or GFP lentivirus particles with 5 mg/ml polybrene (sigma) at a multiplicity of infection (MOI) of 100. The medium was changed after 12 h. After transfection, medium containing FGF2 lentivirus was removed and USC culture medium was added. Three days after transfection, USCs were passaged.

### Endothelial cell differentiation in vitro

To determine the effect of FGF2 on the endothelial differentiation of USCs, we identified the endothelial differentiation of USCs and USCs-FGF2 growing in USC culture medium by immuofluorescence. USCs and USCs-FGF2 induced by endothelial basic medium 2 (EBM2, Lonza) served as a positive control [28]. USCs and USCs-FGF2 of P3 were seeded at a density of 5000 cells/cm^2^ on fibronectin coated dishes (2 μg/cm^2^) and allowed to grow for 2 days to reach a sub-confluent level. EBM2 was used for induction of endothelial differentiation as previously reported [31], [32]. Normal USC medium was also used for both kinds of cells as the study design section describes. These cells were cultured for 9 days. At the conclusion of the treatment period, cells were fixed for the endothelium specific marker (CD31 and vWF) immunofluorescence assay. To evaluate the differentiation efficiency, five fields of 400X magnification images were analyzed by two independent blinded observers. The number of cells expressing CD31 and vWF (magnificantion at 400X) were counted and presented as the percentage of positive cells/total cells.

### Type 2 diabetic erectile dysfunction established in a rat model

After a one-week acclimatization period, 65 rats were fed with a high-fat diet (HFD) for 4 weeks, followed by one dose of streptozotocin (STZ, 35 mg/kg) via intraperitoneal injection [33]. Random blood glucose levels were determined 3 days after STZ injection by tail tip snipping with a glucose meter (Johnson & Johnson, One Touch). Blood glucose levels higher than 300 mg/dl were regarded as diabetic. Body weight was measured every 4 weeks starting with day 1 of HFD administration. To determine insulin resistance of these diabetic rats, an intraperitoneal insulin challenge test was conducted at 6 weeks after STZ injection as reported [33]. One dose of 1 IU/kg bovine insulin in phosphate-buffered solution (PBS) (1 IU/ml) was administered by intraperitoneal injection and blood glucose was tested at 0, 15, 30, 45, 60, 90 and 120 minutes after insulin injection to obtain a blood glucose response curve. The glucose levels are presented as percentages of blood glucose level before insulin injection (baseline). Serum lipid panels were examined at the same time by the Biochemistry Laboratory of the First Affiliated Hospital of Sun Yat-sen University. Apomorphine (APO, 100 μg/kg) (Sigma) was used to identify the ED rats at 7 weeks according to Heaton's method and as previously reported [6], [34]. Rats that did not present erectile response to APO subcutaneous injection were considered to have developed ED. Fifty five rats that developed type 2 diabetic erectile dysfunction were randomly divided into five groups (G2–G5). The remaining 10 non-impotent rats were abandoned.

### In vivo implantation

Eight weeks after STZ injection, the diabetic ED rat model was established following an apomorphine screen. All rats in groups G2–G5 received an injection of 0.2 ml PBS, USCs (1×10^6^ in 0.2 ml PBS), Lenti-FGF2 (1×10^6^ infectious units, 0.2 ml) or USCs-FGF2 (1×10^6^ in 0.2 ml PBS) respectively.

Cultured USCs (P3) and USCs-FGF2 (P3) were trypsinized and then resuspended in PBS for implantation.

Rats were anesthetized with sodium pentobarbital (30 mg/kg, IP). After the penis was exposed, a rubber band was constricted around the base of penis for the implanted cells to be easily delivered [10], [35]. The cell suspension was injected into the cavernosum tissue at the middle of the corpus. The needle was left in place for one minute to allow diffusion of the injected cells from needle into the cavernosum tissue. The elastic band was removed one minute after the injection.

### Determination of erectile function

Intracavernuous pressure (ICP)/Main arterial pressure (MAP) was used to determine erectile function 28 days after intracavernous injection. All animals were anesthetized with sodium pentobarbital (30 mg/kg, IP). A PE-50 catheter filled with 250 units/ml heparinized saline was cannulated to the left carotid artery and connected to a pressure transducer (Taimeng, Chengdu, China) to monitor the arterial blood pressure (ABP). A low abdominal incision was used to expose the right major pelvic ganglion and identify the ipsilateral cavernous nerve (CN). The right corporal body was cannulated with a 23-G butterfly needle to measure the ICP. Continual monophasic rectangular pulses generated by a signal generator (Taimeng, Chengdu, China) were used to stimulate the right CN via a bipolar stainless steel hook electrode. Stimulus parameters were 5 V, 20 Hz, pulse width 0.2 ms, and 60 second duration. The ICP and ABP were recorded at a rate of 10 samples/second by using a sensor input module (BL-420E+, Taimeng, Chengdu, China) connected to a computer with BL_NewCentury 2.0 software (Taimeng, Chengdu, China).

### Histological and immunofluorescence analysis

The rat penile tissues were harvested at days 7 and 28. The implanted USCs were identified by GFP with fluorescence microscopy. The tissues from both days 7 and 28 were stained with endothelial markers (CD31, VEGF). The tissues from day 28 were stained with endothelial functional protein (eNOS), smooth muscle specific markers (desmin and smoothelin), and observed with fluorescence microscopy. Hematoxylin and eosin staining (H&E staining) was performed in tissues of days 7 and 28 to determine whether an inflammatory response occurred. For the semi-quantitative assessment of eNOS, desmin and smoothelin markers, five fields of 400X magnification images were analyzed by two independent blinded observers. The number of cells expressing eNOS, desmin and smoothelin (magnificantion at 400X) were counted and presented as the percentage of positive cells/total cells in the corpora cavernosa. Masson's trichrome-staining was used to determine the ratio of cells to collagen within the cavernous tissues at 28 days.

For immunostaining, tissues were fixed in cold 2% formaldehyde followed by overnight immersion in buffer containing 30% sucrose. The specimens were then embedded in optimum cutting temperature compound (O.C.T.; Sakura Finetec, Torrance, CA) and frozen sections were processed at 8 μm. After incubation with 3% goat serum for 1 hour, tissues were incubated overnight at 4°C with a primary antibody (Table S1) of endothelial markers (CD31, VEGF, eNOS), and smooth muscle specific markers (desmin and smoothelin), followed by a 1-h immersion in a 1∶300 dilution of the secondary antibody conjugated with Alexa-594 Fluor (goat anti mouse, goat anti rabbit, Invitrogen, Carlsbad, CA, USA). Nuclear staining was performed with 4′,6-diamidino-2-phenylindole (DAPI; Cell Signaling, MA, USA).The tissues used for Masson's trichrome and H&E staining were fixed in fresh 4% paraformaldehyde, dehydrated by an ethanol gradient and then embedded in paraffin. Sections were cut at 5 μm and stained according to Masson's trichrome-staining protocol for connective tissue and smooth muscle histology.

### Western blotting

Lyaste was separated by 10% sodium dodecyl sulphate gel (15∼30 μg/lane). Mouse anti CD31 or mouse anti VEGF antibody was applied respectively after transferring proteins to a nitrocellulose membranes. β-actin was used as loading control. Signals were obtained in the linear range of detection and quantified on a quantity one Bio-imaging System. Data was presented as the relative density of each protein compared to β-actin.

### Statistical analysis

Data is reported as mean ± standard (SD) and analyzed by SPSS 20.0. χ^2^ test in order to compare the difference of the CD31 expressing ratio in vitro. Blood glucose, ICP, ICP/MAP ratio and western blot analysis among the five groups were compared by using one-way ANOVA followed by a Student-Newman-Keuls post hoc comparison. We consider differences significant at P values ≤0.05.

## Results

### Measurement of CD markers and paracrine factors of USCs in vitro

**Figure 1:**
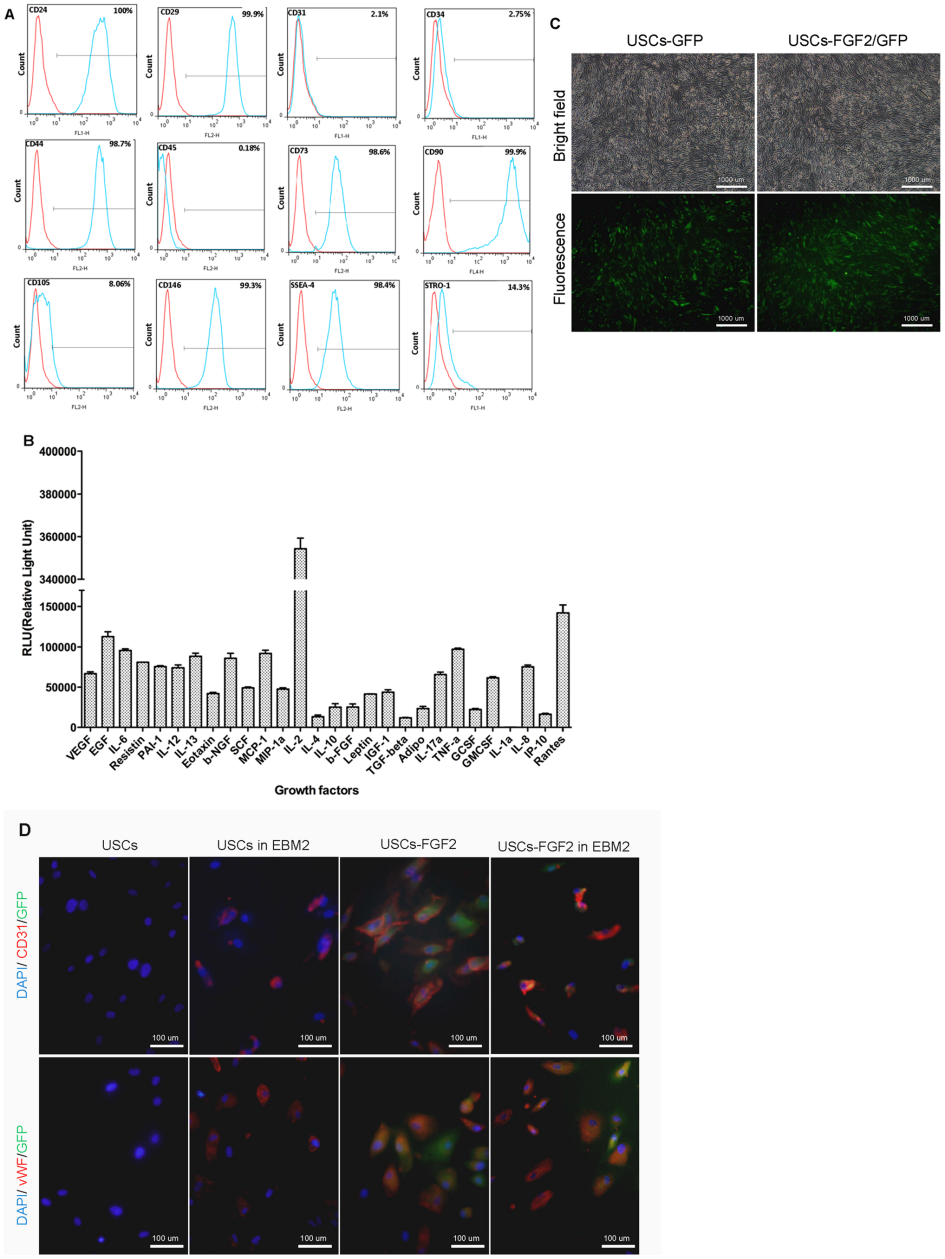
Characteristic of human urine-derived stem cells in vitro. (**A**) Flow cytometric analysis revealed that USCs (p3) display mesenchymal stem cell surface markers (CD24, CD29, CD44, CD73, CD90, CD105, CD146, SSEA4 and STRO-1), but were negative for hematopoietic stem cell markers (CD31, CD34, CD45). (**B**) USCs secreted series proangiogenic growth factors. (**C**) USCs displayed green fluorescence after over-expressing GFP or FGF2/GFP. (**D**) Triple immunofluorescence staining (green-GFP, red-CD31/vWF, blue-DAPI for nuclear staining) for USCs or USCs-FGF2 cultured in normal USC medium or endothelial induction medium (EBM2).

USCs (*p*3) were strongly positive for MSC markers CD24, CD29, CD44, CD73, CD90, CD 146, and SSEA-4, weakly positive for CD105, STRO-1, and negative for the hematopoietic stem cell markers CD31, CD34 and CD45 (Fig. 1A).

Moreover, the USCs (*p*3) could secrete series cytokines including proangiogenic growth factors VEGF, bFGF (FGF2), PDGF and immunomodulatory factors IL-8, IL-10 into the supernatant (Fig. 1B).

### Stable FGF2 expression of USCs

Forty-eight hours after the FGF2/GFP or GFP plasmid was transduced into USCs via lentivirus, the transduction efficiency reached >95% (percentage of cells displaying GFP) (Fig. 1C).

### Differentiation of USCs into endothelial cells in vitro

After being induced in EBM2, 73% USCs and 72% USCs-FGF2 displayed endothelial marker CD31 expression, and 69% USCs and 67% USCs-FGF2 displayed endothelial marker vWF expression (Fig. 1D, Table 1). Without being induced in EBM2, none of the USCs expressed endothelial markers; however, 65% and 66% of USCs-FGF2 expressed CD31 and vWF, respectively, after transfection with FGF2 (compared with USCs or USCs-FGF2 induced by EBM2 P>0.05, Fig. 1D, Table 1). Above data indicates that USCs possess high potency to efficiently give rise to endothelial cells. When grown in normal USC medium, there is a significant difference between USCs and USCs-FGF2 (P<0.01). The increased expression of CD31 and vWF in USCs-FGF2 indicate an effect of genetic modification with FGF2 on promoting the endothelial differentiation of USCs. (Fig. 1D). Endothelial differentiated USCs-FGF2 did not require an extra pro-angiogenic induction condition.

**Table 1:** Endothelial differentiation efficiency of inducted USCs in vitro.

	USCs (%)	USCs in EBM2 (%)	USCs-FGF2 (%)	USCs-FGF2 in EBM2 (%)
CD31	0	73	65	72
vWF	0	69	66	67

### ED rat model of type 2 diabetes mellitus

**Figure 2:**
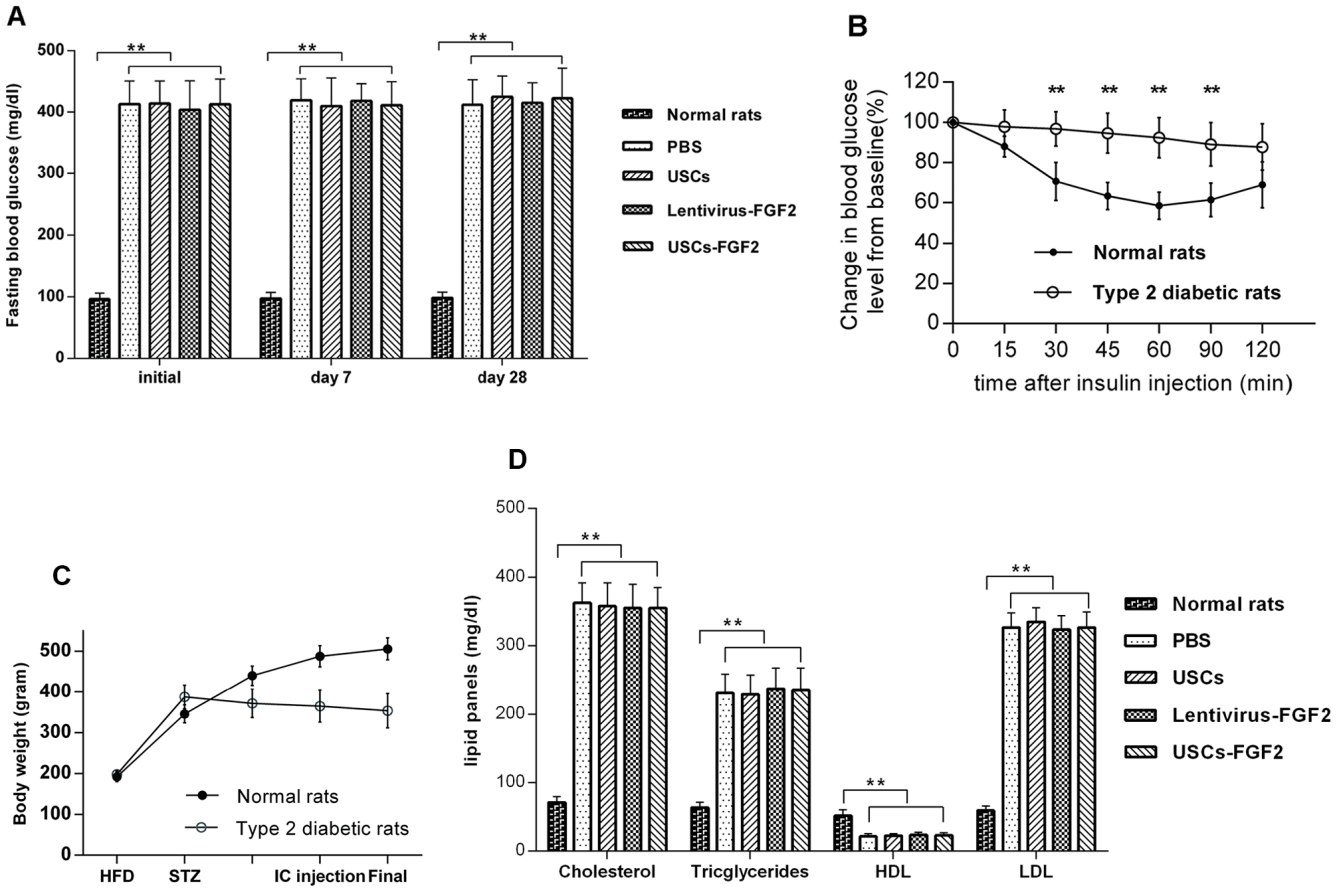
Blood glucose, insulin challenge test, growth curves, and serum lipid panels of the rat. (**A**) Blood glucose levels were significantly higher in all type 2 diabetic rats each time period compared to age-matched non-diabetic controls. (**B**) Insulin challenge test carried out 6 weeks after STZ injection. Compared to normal rats, type 2 diabetic rats lacked response to insulin. (**C**) Body weights remained stable in type 2 diabetic rats. (**D**) The levels of serum triglycerides, total cholesterol, and LDL significantly increased but HDL level significantly declined in the type 2 diabetes rats. (**P<0.01); LDL  =  low-density lipoproteins; HDL  =  high-density lipoproteins.

Three days after intra-peritoneal injection with STZ, blood glucose levels significantly increased in type 2 diabetic rats (G2–G5) when compared to those in the age-matched non-diabetic controls (p<0.01, Fig. 2A). No significant difference in blood glucose levels was noted among the test groups (G2–G5). During the insulin challenge test, blood glucose levels declined significantly in normal rats at 15, 30, 45, 60, 90 and 120 minutes after intraperitoneal injection with insulin. Compared to normal rats, type 2 diabetic rats lacked a response to insulin (P<0.01, Fig. 2B). After STZ injection, the body weights remained stable in type 2 diabetic rats (Fig. 2C). The levels of serum triglycerides, total cholesterol, and low-density lipoproteins (LDL) significantly increased but high-density lipoprotein (HDL) levels significantly declined in the type 2 diabetes rats (P<0.01, Fig. 2D). Among type 2 diabetic rats within the four different treatments, lipid panels were not significantly different.

### Assessment of erectile function

**Figure 3:**
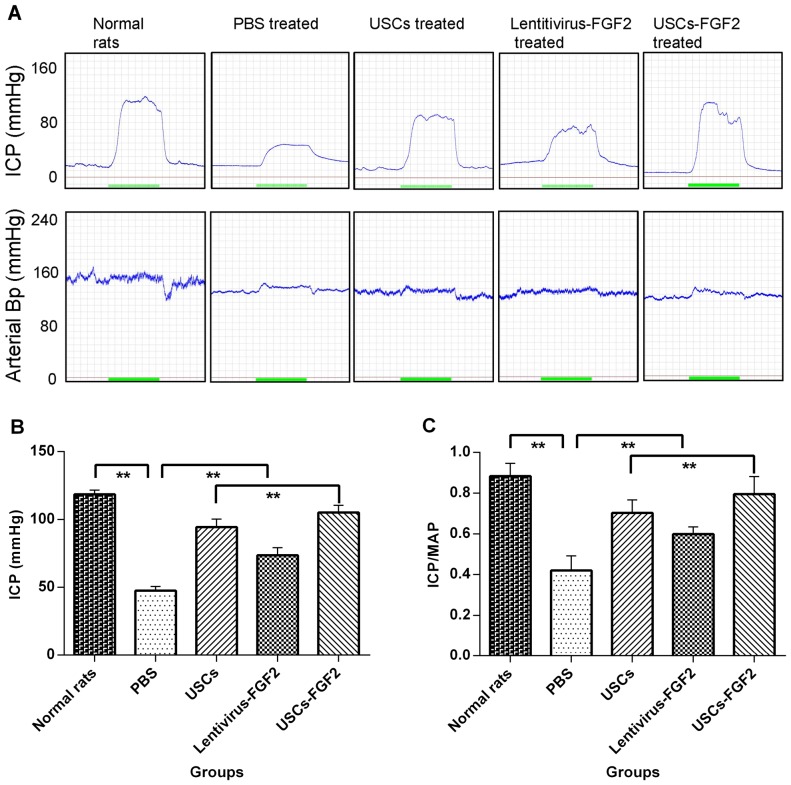
USCs or USCs-FGF2 improved erectile function in type 2 diabetic rats. (**A**) Representative ICP and ABP tracing responses to the stimulation of the cavernous nerve (5 V, 20 Hz, and 60 s duration) in age-matched normal rats or type 2 diabetic rats at 4 weeks after intracavernous injection of PBS or lentivirus-FGF2 or USCs or USCs-FGF2. (each group: n = 10). (**B**) The effects of treatment with USCs or FGF2 or USCs-FGF2 on the increase of ICP. (**C**) The ratio of total ICP to MAP were calculated for each group. ** p<0.01; ICP  =  intracavernous pressure; MAP  =  mean arterial blood pressure; ABP  =  arterial blood pressure.

Representative ICP and ABP tracing responses to the stimulation of the cavernous nerve (0.2 ms, 5 V, 20 Hz, and 60 seconds duration) were recorded in all groups 4 weeks after intracavernous injection (Fig. 3A). Control rats displayed normal ICP curves and significant higher ICP and ICP/MAP ratios than PBS treated type 2 diabetic rats (P<0.01). After treatment with USCs-FGF2 (G5), ICP and the ICP/MAP ratios were significantly increased (P<0.01) in diabetic rats, although the values were still significantly lower than that in normal rats (P<0.01). The ICP in USC (G3) or Lentivirus-FGF2 (G4) treated rats was also significantly higher than in PBS treated rats (P<0.01), but significantly lower than that in the USC-FGF2 rats (G5) (P<0.01). (Figs. 3B, 3C).

### Detection of survived USCs after injection

**Figure 4:**
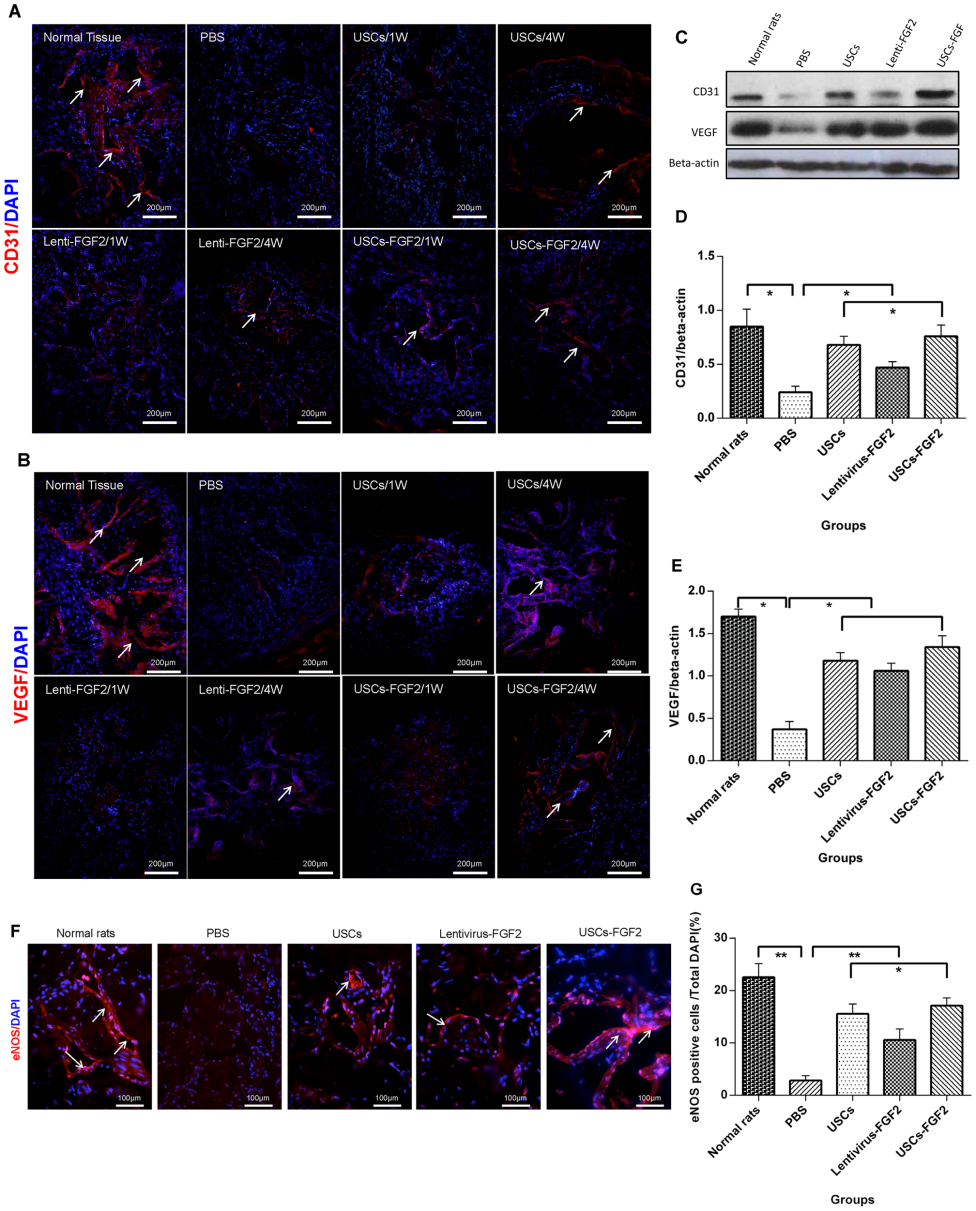
USCs or USCs-FGF2 increased expression of CD31, VEGF and eNOS in corpora cavernosa. Immunofluorescence staining displayed expression of CD31 (**A**) and VEGF (**B**) (white arrow represents specific staining) was increased in USCs-FGF2 or USCs or lentivirus-FGF2 groups in type 2 diabetic rats after 4 weeks. Non specific staining of GFP was found in each group. Western blotting analysis (**C**) and quantitative analysis for Western blotting (**D, E**) confirmed the significantly increased expression of CD31 and VEGF. Immunofluorescence staining (**F**) and hemiquantitative analysis (**G**) showed USCs-FGF2 or USCs or lentivirus-FGF2 increased the number of cells expressing eNOS. * P<0.05, ** P<0.01.

Injected USCs were tracked in penis tissue sections by green fluorescence. No green florescence was observed among corporal sinusoids of all groups on either day 7 or 28. (Figs. 4A, 4B).

### Expression of endothelial marker (CD31) in the corpora cavernosa tissue

Number of cells expressing CD31 (Fig. 4A) or the amount of endothelial proteins (CD31) (Figs. 4C, 4D) was significantly lower within the corpora cavernosa in PBS-treated ED rats, when compared to normal rats (P<0.05) in immunofluorescence staining and western blot analysis. However, endothelial content within the cavernous tissue was significantly restored after USC-FGF2 injection (G5) (P<0.05), while there was only a partially recovery in endothelial content in USC or lentivirus-FGF2 injection alone.

### Expression of VEGF in vivo

Expression of VEGF within the corpora cavernosa was significantly lower in PBS-treated ED rats (G2) than that in the age-matched G1 control (p<0.05), when assessed with immunofluorescence staining and Western blot. After intracavernous injection with USCs-FGF2, expression of VEGF within corpora tissue significantly increased but was lower than the age-matched G1 controls (p<0.05). In addition, USC or lentivirus-FGF2 treated ED rats also showed a significant increase of VEGF expression when compared to PBS treated ED rats but results were lower than in USC-FGF2 treated rats (p<0.05). (Figs. 4B, 4C, 4E).

### Expression of endothelial nitric oxide synthase (eNOS)

The number of the cells expressing eNOS within corpora tissue significantly decreased in type 2 diabetic impotent rats (G2), compared with age-matched controls (G1), as confirmed by immunofluorescence staining with semi-quantitative analysis (P<0.01, Figs. 4F, 4G). USCs-FGF2 significantly increased the number of cells expressing eNOS (P<0.01, Figs. 4F, 4G). The number of cells expressing eNOS within the corpora tissue in USC (G3) or lentivirus-FGF2 (G4) treated rats was significantly higher than those in PBS treated rats (G2) (P<0.05), but significantly lower than those in USC-FGF2 rats (G5) (P<0.05, Figs. 4F, 4G).

### Expression of smooth muscle marker and cell to collagen ratio in corpora cavernosa

**Figure 5:**
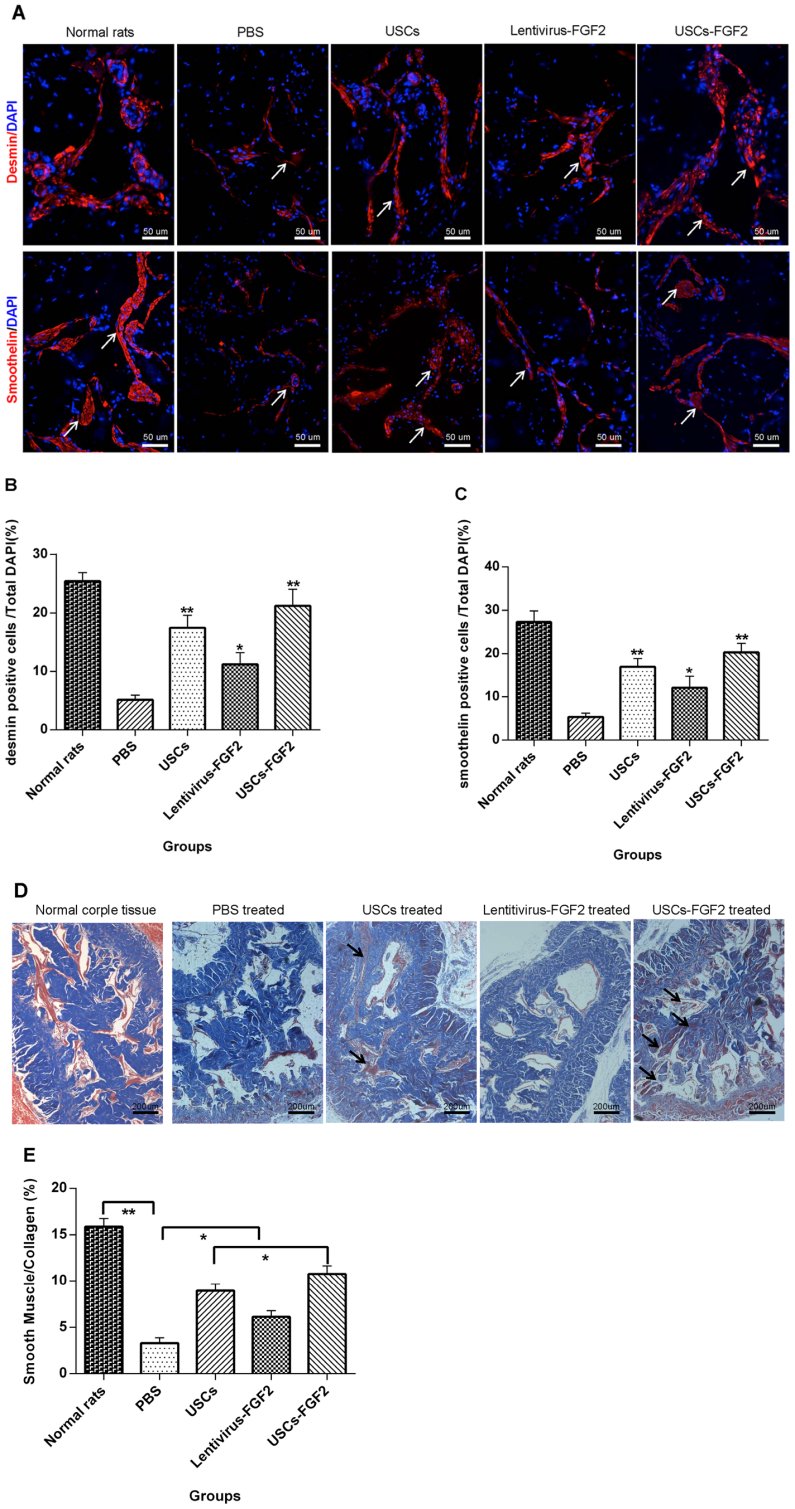
Smooth muscle cells/collagen ratio increased in USCs or USCs-FGF2 treated group. smooth muscle specific markers (desmin and smoothelin) assessed by immunofluorescence (**A**); Semi-quantitation evaluation of number of cells expressing desmin (**B**), and smoothelin (**C**) (compared with PBS treated group, * P<0.05, ** P<0.01); Masson's Trichrome staining (**D**); Semi-quantitation evaluation of smooth muscle cells/collagen ratio (**E**). * P<0.05, ** P<0.01.

Immunofluorescence staining with semi-quantitative analysis showed that the cell numbers expressing smooth muscle markers (desmin and smoothelin) was significantly decreased within the corpora cavernosa in PBS treated ED rats when compared to those in the age-matched G1 control (P<0.01, Figs 5A, 5B, 5C). Intracavernous injection with either USCs-FGF2 or USCs significantly increased the number of cells expressing smooth muscle markers within the corpora tissue (P<0.01, Figs 5A, 5B, 5C). The number of cells expressing smooth muscle markers within the corpora tissue in lentivirus-FGF2 treated rats were significantly higher than those in PBS treated rats (P<0.05, Figs 5A, 5B, 5C), while significantly lower than those in USC-FGF2 or USC treated rats (P<0.05, Figs 5A, 5B, 5C).The cell/collagen ratio, confirmed by Masson's trichrome-staining (Fig. 5D), was also significantly decreased in the PBS treated group (P<0.01). After receiving intracaverous injection of USCs-FGF2 the cell/collagen ratio significantly increased (P<0.05, Figs. 5D, 5E). USC or lentivirus-FGF2 injection had a slightly lower (p<0.05, Figs. 5D, 5E) improvement in cell/collagen ratio.

### H&E staining

**Figure 6:**
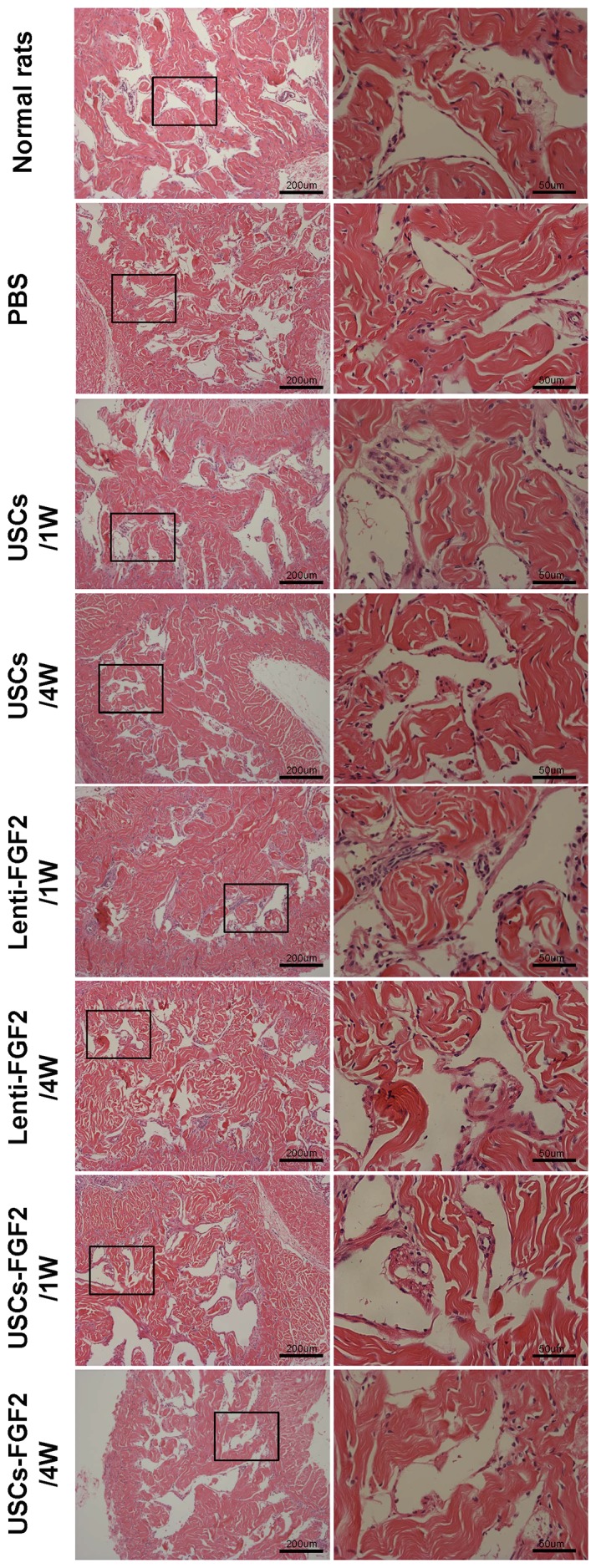
Rat penile sections stained with hematoxylin and eosin. Neither lymphocytic infiltrates nor inflammatory response was evident in the corpora cavernosa of rats injected with PBS, USCs, FGF2 lentivirus particles or USCs-FGF in all experiments.

Neither lymphocytic infiltrates nor inflammatory response was evident within the corpora cavernosa in all rat test groups with H&E staining on days 7 and 28 after cell injection (Fig.6).

### Blood glucose concentration after intracavernous injection

Blood glucose concentrations were still significantly higher in the four treatment groups (G2–G5) than in the G1 control group (P<0.01), though they were not significantly different among each treatment group on days 7 and 28 after cell injection (Fig. 2A).

## Discussion

Pathophysiology of diabetes mellitus-related ED is associated with endothelial dysfunction at an early stage [8], [36]. Impaired vasodilatory response is attributed to nitro oxide inhibition, smooth muscle cell dysfunction, and chronic nerve damage due to such long-term hyperglycemia [3], [4], [5], [6], [7]. This damage remains difficult to treat medically despite advances in pharmacotherapeutic approaches [8]. However, cell-based therapy is a promising treatment for diabetes-related ED, particularly in the initial stages. Most studies have focus on MSCs derived from bone marrow or fat tissues [11], [12], [13], [14], [37] for diabetic ED. In this study, we demonstrated that intracavernous injection of USCs or USCs-FGF2 significantly improved the endothelial functional protein (eNOS) expression, restored the number of cells expressing endothelial marker (CD31) and increased ICP to enhance erectile function in the diabetic ED rodent model. USCs can secrete pro-angiogenic trophic factors and immune-modulatory factors in vitro, which might play a part in the recovery of erectile function in this study. Although USCs can differentiate into endothelial cells by modification with FGF2 and without induction of growth factors in vitro, we failed to track the injected USCs within the penis tissue.

The impaired VEGF signaling pathway in the corpora cavernosa is another key contributing factor to diabetic endothelial dysfunction [6], [38]. In this experiment, we found USCs-FGF2 increased endogenous VEGF protein expression, indicating the FGF2 modification may repair the VEGF signaling pathway in the corpora cavernosa of diabetic ED rats. FGF2 is a member of the fibroblast growth factor family, which binds heparin and possesses broad mitogenic and angiogenic activities. FGF2 induces VEGF expression in vascular endothelial cells forming capillaries through autocrine and paracrine mechanisms [39]. It is also reported that the FGF2/VEGF axis participates in FGF2-mediated angiogenesis [40]. We found that FGF2 delivery by USCs to the penis of impotent diabetic rats may also increase endogenous VEGF protein expression in the corpus cavernosum. The increased VEGF is sufficient for organ homing of circulating mononuclear myeloid cells, including recruited bone marrow-derived circulating cells (RBCCs) [41]. RBCCs may further enhance in situ proliferation of endothelial cells via secreting pro-angiogenic activities distinct from locally induced activities [41].

Aside from the endothelium, smooth muscle is a second key structural component for penile erection. A decreased smooth muscle/total collagen ratio was reported in type 2 diabetic ED rats [33], and may contribute to ED by decreasing the ability of the sinusoids to expand, thereby resulting in veno-occlusive dysfunction [42]. In this study, the smooth muscle/total collagen ratio was also increased after intracavernous administration of USCs or USCs-FGF2. The restored smooth muscle/total collagen ratio may relate to the relaxation and nutrition of endothelium cells and smooth muscle cells in the corpora cavernosa.

As human cells were injected into rats, immunological rejection should be considered. The immunomodulatory and immunosuppressive property of MSCs is believed to permit their allogeneic or even xenogeneic transplantation into immunocompetent recipients without the use of immunosuppressants [43]. In 2000, Leichty et al. [44] reported that human Bone marrow MSCs survived for 13 months, exhibited tissue engraftment, and underwent site-specific cell differentiation after transplantation into immunocompetent fetal sheep. The rat model was also demonstrated to avoid immunological rejection when receiving transplantation of human ADSCs [45]. As a kind of MSCs, USCs also possess immunomodulatory property in consistently expressing a whole panel of MSC surface markers, but not the main histocompatibility complex (MHC) classII[32]. In addition, USCs inhibit peripheral blood mononuclear cell (T and B cell) proliferation and secrete the immunoregulatory cytokines interleukin (IL)-6 and IL-8 [46]. Furthermore, histological analysis did not show immune reaction or inflammatory response occurrence within the injected sites in the penile tissue.

There are certain limitations in this work. Trans-differentiation and paracrine potential are often considered the main roles of MSCs in tissue repair. However, we failed to track the injected USCs within corporal sinusoids, indicating that trans-differentiation of USCs did not play a role to improve diabetic ED in this study. The most likely reason is that a majority of implanted individual cells rapidly flowed into the blood circulation system after injection. To deliver the cells more efficiently into the cavernous tissue, we used a thin rubber band to tie the penis base before injection, and then the rubber band was removed one minute after injection. Although the implanted cells were not systematically detected, the cells were most likely alive and secreted the pro-angiogenic growth factors and cytokines needed to recruit the resident cells and contributing to the improvement of diabetic ED and impaired cavernous tissues. In addition, cell labeling with GFP is also not yet ideal to track the implanted cells. Because of the interference of autofluorescence within the host tissue, GFP detection can seem like “seeing the wood through the trees” [47]. Potential biochemical sources of autofluorescence include flavin, NAD(P)H, Lipofuscins, advanced glycation end-products (AGE), collagen and elastin [47]. These sources of autofluorescence also reduce the contrast, affecting the clarity of GFP labeled structures in fluorescence microscope imaging. To track the transplanted stem cells, more reliable and efficient labeling techniques are needed. In addition, injection of 3D microspheres with seeded cells might prevent cell flashing away after a rubber band is released, which will enhance cell attachment, survival, ingrowth, and differentiation of stem cells by increasing size of cell aggregates [48]. To improve the therapeutic effect of USCs or USCs-FGF2 and understand their exact mechanism, further investigation in these areas is needed.

## Conclusions

In this study, we have demonstrated that USCs or USCs genetically-modified with FGF2 enhance the expression of endothelial cell markers, smooth muscle contents, and improve neurogenic-mediated erectile responses in type 2 diabetic ED rats. The outcome of improvement in diabetic ED in a rodent model after administration of USCs or USCs-FGF2 is similar to those in cell therapy with other types of MSCs. Paracrine action of USCs may play an important role in recruiting resident endothelial and smooth muscle cells to participate in tissue repair within the cavernous tissue.

## Supporting Information

Table S1 **Details of antibodies used in this study.**
(DOC)
