# Supplementary material for: Human Urine-Derived Stem Cells Alone or Genetically-Modified with FGF2 Improve Type 2 Diabetic Erectile Dysfunction in a Rat Model
Source: PLoS One. 2014 Mar 24;9(3):e92825. doi: 10.1371/journal.pone.0092825 (PMC3963968; doi:10.1371/journal.pone.0092825)
Supplement: Table S1 — Details of antibodies used in this study. (DOC) [file pone.0092825.s001.doc]

Table S1. Details of antibodies used in this study

|  |  | Antibody Dilution | | |
| --- | --- | --- | --- | --- |
| Primary Antibody | Company/Catalog # | IF | WB | FACS |
| CD24-FITC | BD 560992 |  |  | 20 μl/test |
| CD29-PE | BD 556049 |  |  | 20 μl/test |
| CD31-FITC | BD 560984 |  |  | 20 μl/test |
| CD34-FITC | BD 348053 |  |  | 20 μl/test |
| CD44-PE | BD 550989 |  |  | 20 μl/test |
| CD45-PE | BD 555483 |  |  | 20 μl/test |
| CD73-PE | BD 550257 |  |  | 20 μl/test |
| CD90-APC | BD 559869 |  |  | 5 μl/test |
| CD105-PE | BD 560839 |  |  | 5 μl/test |
| CD146-PE | BD 550315 |  |  | 20 μl/test |
| SSEA-4-PE | BD 560128 |  |  | 20 μl/test |
| STRO1-FITC | Biolegend 340105 |  |  | 2.5 μl/10^6^ cells |
| VEGF | Abcam /ab68334 | 1:50 | 1:1000 |  |
| CD31 | Abcam/ab24590 | 1:50 | 1:500 |  |
| vWF | Abcam /ab6994 | 1:100 |  |  |
| eNOS | Abcam/ab5589 | 1:100 |  |  |
| Desmin | Santa Cruz/SC-7559 | 1:100 |  |  |
| Smoothelin | Abcam/ab8969 | 1:100 |  |  |
